# Exposure of *Lycopersicon Esculentum* to Microcystin-LR: Effects in the Leaf Proteome and Toxin Translocation from Water to Leaves and Fruits

**DOI:** 10.3390/toxins6061837

**Published:** 2014-06-11

**Authors:** Daniel Gutiérrez-Praena, Alexandre Campos, Joana Azevedo, Joana Neves, Marisa Freitas, Remédios Guzmán-Guillén, Ana María Cameán, Jenny Renaut, Vitor Vasconcelos

**Affiliations:** 1 Area of Toxicology, Faculty of Pharmacy, University of Seville, Seville 41012, Spain; E-Mails: dgpraena@us.es (D.G.-P.); rguzman1@us.es (R.G.-G.); camean@us.es (A.M.C.); 2 Interdisciplinary Centre of Marine and Environmental Research (CIIMAR/CIMAR), Porto 4050-123, Portugal; E-Mails: joana_passo@hotmail.com (J.A.); joana.t.neves@gmail.com (J.N.); 3 Polytechnic Institute of Porto, Escola Superior de Tecnologia da Saúde do Porto, Research Center in Environment and Health (CISA), Gaia 440-330, Portugal; E-Mail: maf@estsp.ipp.pt; 4 Department of Environment and Agro-biotechnologies (EVA), Centre de Recherche Public-Gabriel Lippmann, Belvaux L-4422, Luxembourg; E-Mail: renaut@lippmann.lu; 5 Department of Biology, Faculty of Sciences of the University of Porto, Porto 4169-007, Portugal; E-Mail: vmvascon@fc.up.pt

**Keywords:** *Lycopersicon esculentum*, microcystin, phytotoxicity, bioaccumulation, proteomics

## Abstract

Natural toxins such as those produced by freshwater cyanobacteria have been regarded as an emergent environmental threat. However, the impact of these water contaminants in agriculture is not yet fully understood. The aim of this work was to investigate microcystin-LR (MC-LR) toxicity in *Lycopersicon esculentum* and the toxin accumulation in this horticultural crop. Adult plants (2 month-old) grown in a greenhouse environment were exposed for 2 weeks to either pure MC-LR (100 μg/L) or *Microcystis aeruginosa* crude extracts containing 100 μg/L MC-LR. Chlorophyll fluorescence was measured, leaf proteome investigated with two-dimensional gel electrophoresis and Matrix Assisted Laser Desorption Ionization Time-of-Flight (MALDI-TOF)/TOF, and toxin bioaccumulation assessed by liquid chromatography-mass spectrometry (LC-MS)/MS. Variations in several protein markers (ATP synthase subunits, Cytochrome b6-f complex iron-sulfur, oxygen-evolving enhancer proteins) highlight the decrease of the capacity of plants to synthesize ATP and to perform photosynthesis, whereas variations in other proteins (ribulose-1,5-bisphosphate carboxylase/oxygenase large subunit and ribose-5-phosphate isomerase) suggest an increase of carbon fixation and decrease of carbohydrate metabolism reactions in plants exposed to pure MC-LR and cyanobacterial extracts, respectively. MC-LR was found in roots (1635.21 μg/kg fw), green tomatoes (5.15–5.41 μg/kg fw), mature tomatoes (10.52–10.83 μg/kg fw), and leaves (12,298.18 μg/kg fw). The results raise concerns relative to food safety and point to the necessity of monitoring the bioaccumulation of water toxins in agricultural systems affected by cyanotoxin contamination.

## 1. Introduction

Cyanotoxins are a diverse group of natural substances produced by several species of cyanobacteria. One of the groups of cyanotoxins of most concern for human health are Microcystins (MCs), as they are present in a wide range of habitats and the possibility of exposure exists via skin contact or by ingestion of contaminated food and water.

MCs are hepatotoxic cyanotoxins produced by several genera of cyanobacteria such as *Microcystis*, *Anabaena*, *Plankthotrix*, *Nostoc*, *Anabaenopsis*, and *Hapalosiphon* [1]. There are over 80 different MC congeners [2], consisting of cyclic heptapeptides containing both l- and d-amino acids and a hydrophobic C20 d-amino acid ADDA (3-amino-9-methoxy-2,6,8-trimethyl-10-phenyldeca-4,6-dienoic acid) [3]. The most common, and also the most extensively studied, MCs are MC-LR, MC-RR, and MC-YR, with MC-LR being the most toxic [4]. MCs’ main molecular targets are protein phosphatases PP1 and PP2A to which they bind covalently. MCs also induce oxidative stress through the production of reactive oxygen species (ROS) both *in vivo* [5,6] and *in vitro* [7,8]. In addition, MCs can also act as tumor promoters, and MC-LR has been considered as a potential carcinogen to humans (group 2B) by the International Agency for Research on Cancer (IARC) [9,10].

MCs can be found mainly inside the producer cells (75%) but also dissolved in the aqueous media at concentrations dependent on cyanobacterial decay [11,12]. Although most of cyanobacterial blooms occur in open aquatic systems such as oceans, rivers, lakes, ponds, *etc.*, they can also appear in waters intended for plant irrigation and agriculture. For this reason, it has been hypothesized that MCs can be accumulated in edible plant tissues, and thus being a potential risk to food safety and to human health via diet. In this regard, MCs were found in field grown water chestnuts at concentrations up to 7 μg/kg dry weight (dw) [13], in tomato fruits collected from the field (1.16 μg/kg dw) [14] and in several other vegetable crops irrigated using contaminated groundwater (70–1200 μg/kg fresh weight of MCs) [15]. Laboratory studies have shown that clover, rape, and lettuce plants accumulated 0.2–1.45 mg/kg dw MCs when exposed to 2.1 mg/L MCs for 15 days [16]. Moreover MCs’ average bioconcentration factors (BCF) of 3.6 and 8.4 were calculated in shoots and roots from 11 agricultural species [17]. The adverse effects of cyanotoxins in crops can also reverberate in lower growth, lower productivity rates, and lower quality products. In this regard, toxicity studies have shown that different concentrations of MCs and crude cyanobacteria cell extracts inhibit *Sinapis alba* growth [18]. Growth inhibition has also been reported in other crops [19,20]. MCs also have a negative impact on seed germination and root development [20,21,22,23] and have recently been shown to inhibit nodulation and Rhizobia growth with the potential for impairment of nitrogen uptake in leguminous crops [24,25]. Moreover evidence was collected at the molecular level, which highlighted the physiological stress of the plants after exposure to MCs [20,24,26,27,28,29].

In line with the current knowledge on the effects of algal toxins in terrestrial plants, this work aims to gather further insights regarding the toxic effects of cyanobacterial MC-LR in *Lycopersicon esculentum* (tomato) and to examine MC-LR accumulation in the different tissues of this important crop. Our hypothesis was that MC-LR could be taken up by roots and transferred to leaves and fruits of the plants. To this end, adult tomato plants exposed to *M. aeruginosa* cell crude extracts and to pure MC-LR (100 μg/L) were analyzed in regard to the proteome, using two-dimensional gel electrophoresis (2DE) and mass spectrometry (MS), and for MC-LR accumulation in the different tissues using liquid chromatography coupled with MS (LC-MS).

## 2. Results

### 2.1. Photosynthetic Efficiency

**Figure 1:**
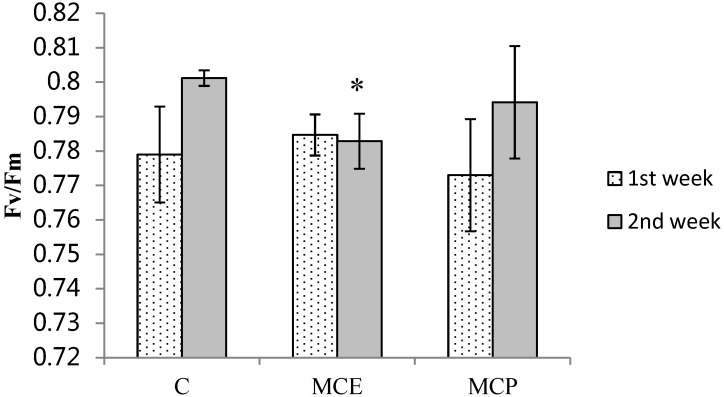
Chlorophyll fluorescence in *Lycopersicon esculentum* leaves. Control group (C), group treated with *M. aeruginosa* crude extracts (MCE) and group treated with pure microcystin-LR (MC-LR) (MCP). Significant differences relative to control, with *p* < 0.05 (*).

Maximum fluorescence yield of Photosystem II (Fv/Fm) was measured in control plants and plants treated with *M. aeruginosa* crude extracts (MCE) and MC-LR (MCP) to assess photosynthetic efficiency. Data show the decrease of Fv/Fm in plants treated for 2 weeks with MCE (Figure 1), which could be related to an impairment of photosynthesis in this group of plants. In contrast pure MC-LR did not affect photosynthesis.

### 2.2. Protein Expression in Tomato Leaves

**Figure 2:**
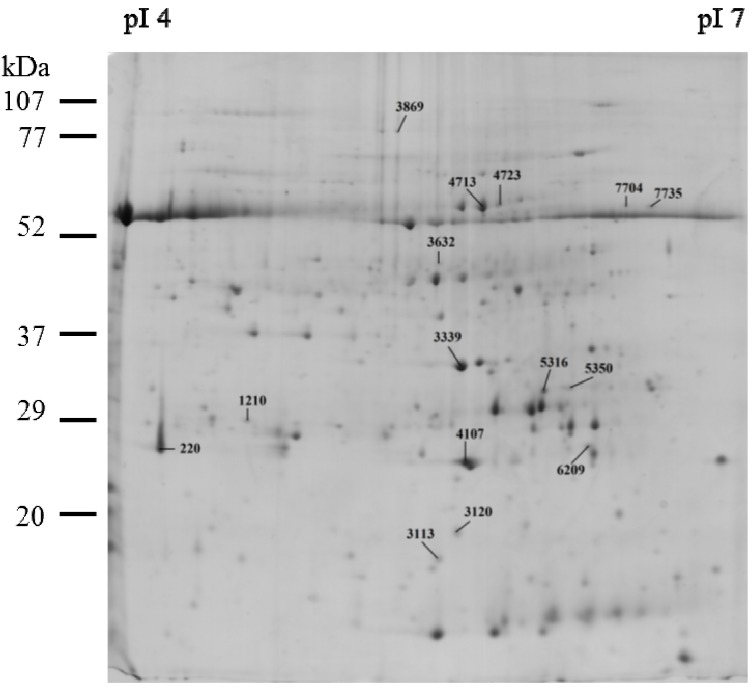
Two dimensional electrophoresis (2DE) map of the differentially expressed proteins identified by Matrix Assisted Laser Desorption Ionization Time-of-Flight (MALDI-TOF)/TOF mass spectrometry, in *Lycopersicon esculentum* leaves. 2DE gels were loaded with 300 μg of protein. Isoelectric focusing was carried out in 17cm immobiline IEF gel strips, pH range 4–7. The second dimension sodium dodecyl sulfate polyacrylamide gelelectrophoresis (SDS-PAGE) was performed in 12% (w/v) polyacrylamide gels. Gels stained with Colloidal Coomassie G-250.

Protein expression was undertaken in the leaves of tomato plants aiming to gather additional insights into the metabolic disturbances induced by *M. aeruginosa* secondary metabolites and MC-LR. Regarding this, a proteomics approach followed consisting of two-dimensional electrophoresis (2DE) and Matrix Assisted Laser Desorption Ionization Time-of-Flight (MALDI-TOF)/TOF. Proteins were separated between pI 4 and 7 and molecular masses between 15 kDa and 80 kDa (Figure 2). Protein profiles were compared between experimental groups (control *versus* MCE and MCP) and quantitative variations evaluated. Eleven proteins were differentially expressed in MCE treated plants, and nine in MCP treated plants, relative to control plants. Functions were assigned to the majority of this group of proteins (15 proteins identified by MALDI-TOF/TOF). The differential expression of the identified proteins in the three experimental groups is summarized in Table 1. The 2DE map of these proteins is shown in Figure 2.

**Table 1:** Differentially expressed proteins in leaves of *Lycopersicon esculentum*. Normalized intensity and standard deviation (SD) of each identified protein in each experimental group, control plants (C), plants exposed to *M. aeruginosa* extracts (MCE) and pure MC-LR (MCP). Statistical differences, with *p* < 0.05 (statistics: stat.).

Biological Function	Protein Name/Accession (NCBI)	SSP	C		MCE		MCP		Stat.
			Mean	SD	Mean	SD	Mean	SD	
ATP synthesis	ATP synthase CF1 epsilon subunit/gi|89280641	3,113	2,161.17	523.64	1,083.10	67.11	1,614.57	289.78	a↑
	ATP synthase CF1 alpha chain/gi|89280620	4,713	16,331.87	415.97	9,004.00	3,492.91	8,123.90	4,598.80	a↓, b↓
	ATP synthase CF1 alpha subunit/gi|28261702	4,723	1,784.37	416.06	892.93	96.02	1,144.53	739.62	a↓
	ATP synthase delta chain, chloroplastic/gi|416681	6,209	2,544.37	344.27	1,369.07	225.35	1,968.50	505.96	a↓
Carbon fixation	Ribulose-1,5-bisphosphate carboxylase/oxygenase large subunit/gi|77798370	7,704	265.55	197.49	503.30	0.00	650.77	158.04	b↑
	Ribulose-1,5-bisphosphate carboxylase/oxygenase large subunit/gi|21634137	7,735	526.30	570.78	906.40	704.28	1331.10	293.83	a↓
Photosynthesis	Cytochrome b6-f complex iron-sulfur subunit, chloroplastic/gi|68566175	3,120	2,037.23	437.05	780.53	432.47	1,609.97	76.46	b↓
	Oxygen-evolving enhancer protein 1, chloroplastic/gi|12644171	3,339	17,411.43	4,066.45	21,763.37	11,695.21	9,877.70	688.13	a↓, b↓
	Oxygen-evolving enhancer protein 2, chloroplastic/gi|350538909	4,107	29,152.87	1,140.43	9,704.93	4,910.78	18,299.77	2,863.23	a↓
Carbohydrate	Ribose-5-phosphate isomerase/gi|225451299	5,316	4,453.07	361.92	2,821.67	319.86	3,672.17	362.55	a↓
Metabolism	Ribose-5-phosphate isomerase/gi|225451299	5,350	1,201.00	247.53	610.03	42.48	899.83	239.29	b↑
Proteolysis	ATP-dependent Clp protease ATP-binding subunit clpA homolog CD4B, chloroplastic/gi|399213	3,869	316.30	0.00	451.53	262.70	686.13	279.57	a↓, b↓
Embryogenesis and stress response	24K germin like protein/gi|58244966	220	18,919.30	5,892.38	6,637.10	948.09	7,986.73	2,663.81	b↓
Uncharacterized protein	Heme-binding protein 2/ gi|225470739	1210	1,193.80	332.38	711.40	223.81	576.47	187.06	b↑
Cytoskeleton	Actin/gi|32186906	3,632	384.07	92.56	1,043.73	1,034.54	1,606.87	731.04	a↑

Notes: (a) Significant variations in protein expression in MCE group relative to control (*p* < 0.05). Down-regulated (↓) and up-regulated proteins (↑); (b) Significant variations in protein expression in MCP group relative to control (*p* < 0.05). Down-regulated (↓) and up-regulated proteins (↑).

Plants exposed to the two treatments showed a decrease in the expression of several subunits of the chloroplastidial ATP synthase (alpha, epsilon, and delta subunits) and of several components of the photosynthesis apparatus (cytochrome b6-f complex iron-sulfur subunit, oxygen-evolving enhancer proteins 1 and 2). The 24K germin like protein also decreased in plants exposed to both treatments relative to control plants (Table 1). On the other hand, *M. aeruginosa* extracts led to the specific inhibition of ribose-5-phosphate isomerase while the pure toxin caused the over-expression of ATP-dependent Clp protease, actin, and ribulose-1,5-bisphosphate carboxylase/oxygenase (rubisco) large subunit, and to the reduction of an uncharacterized protein with a hypothetical heme-binding function (heme-binding protein 2) (Table 1).

### 2.3. Toxin Quantification in Tomato Tissues

Quantification of free MC-LR in plant tissues was accomplished by LC-MS/MS analysis. The area of the peak corresponding to the *m*/*z* 995 (retention time approximately 5.8 min) was calculated based on the linear regression of a standard calibration curve. The presence of the toxin was validated by performing MS/MS on the precursor molecule (*m*/*z* 995) and the detection of at least two of the reference fragment ions with *m*/*z* 375, 553, 599, 866 and 977 (the figure in the supporting material illustrates the chromatograms obtained for several samples and the MS/MS spectra confirming the presence of MC-LR). The toxin was accumulated in fruits and leaves after one week of exposure to MC-LR. MC-LR concentrations in green tomatoes varied between 5.15 and 5.41 μg/kg fw, in mature tomatoes between 10.52 and 10.83 μg/kg fw, while in young leaves (second and third pairs from the apex) the concentration reported was 12,298.18 μg/kg fw (Table 2). However, the toxin levels in fruits decreased during the second week of treatment to below the detection limit of the instrument. Root samples were collected only at the end of the exposure experiment, mass spectrometry analysis showed elevated concentrations of MC-LR in this organ (1635.21 μg/kg fw) (Table 2). Strikingly, MC-LR in roots and leaves was only detected in plants exposed to *M. aeruginosa* extracts, but not to pure MC-LR.

**Table 2:** Concentration of MC-LR determined in the different tomato plant tissues including fruits. Not analyzed (ND); concentrations below the limit of detection of the equipment (LD, 0.58 μg/L).

Tissue	Treatment	MC-LR (μg/kg FW Tissue)	
		Week 1	Week 2
Root	C	ND	<LD
	MCE	ND	1635.21 ± 941.11
	MCP	ND	<LD
Leaves	C	<LD	<LD
	MCE	12,298.18 ± 8962.03	nd
	MCP	ND	<LD
Green tomato	C	<LD	<LD
	MCE	5.41 ± 0.49	<LD
	MCP	5.15 ± 0.93	<LD
Mature tomato	C	<LD	<LD
	MCE	10.52 ± 6.48	<LD
	MCP	10.83 ± 0.94	<LD

## 3. Discussion

### 3.1. Impact of Cyanobacterial Compounds and MC-LR in Plant Metabolism

The proteomic analysis of the leaves of tomato plants exposed to *M. aeruginosa* crude extracts and pure MC-LR have revealed variations in several proteins related with ATP synthesis (ATP synthase CF1 subunits alpha, epsilon, and delta), carbon fixation (ribulose-1,5-bisphosphate carboxylase/oxygenase large subunit), photosynthesis (Cytochrome b6-f complex iron-sulfur subunit, oxygen-evolving enhancer proteins 1 and 2) and carbohydrate metabolism (ribose-5-phosphate isomerase). These protein markers highlight the putative decrease of the capacity of plants to synthesize ATP and to perform photosynthesis. Carbon fixation, however, may be increased upon plant exposure to the pure toxin only, whereas carbohydrate metabolism may be repressed due to MCE exposure. Other cellular processes likely affected by pure MC-LR concern proteolysis (putative increase), stress defense (increase in 24 kDa germin like protein), and cytoskeleton formation. Although significant changes have been reported in major proteins of the primary metabolism, leaf chlorosis and/or necrosis were not observed after 2 weeks of exposure to MC-LR nor any decline in plant growth or yield. Moreover photosynthetic efficiency was reduced only after 2 weeks of exposure to *M. aeruginosa* extracts. Results thus point to a weak toxicity induced by100 μg/L MC-LR and *M. aeruginosa* extracts in this agricultural crop. It should be retained that the *M. aeruginosa* strain LEGE 91094 used in this work produces mainly the variant MC-LR (95%) and MC-LA in a lower percentage (5%), therefore the toxic effects derived from the exposure to the respective cell extracts can be attributed mainly to the MC-LR bioactivity.

Evidence exists that indicates that phytotoxic effects are plant species-, dose-, time-, and life stage-dependent [30,31,32]. Toxicity has been confirmed in laboratory studies for relatively high toxin concentrations, in the range of hundreds to thousands of micrograms per liter. In this regard, seedlings seem to be very sensitive to the exposure to MCs and their development more affected than, for instance, germination. Chen *et al.* (2004) [21] reported that germination of rice and rape was not affected by cyanobacterial extracts containing 120 μg/L MCs. In contrast, seedlings were shown to be sensitive to this toxin concentration. Germination tests performed with four agricultural plants (*Pisum sativum*, *Lens esculenta*, *Zea mays* and *Triticum durum*) revealed that 1600 μg/L total microcystins (MCs) in a cyanobacterial bloom extract inhibited the germination rate of *Pisum sativum* only. However, a substantial inhibition of seedling growth was reported for all plants tested, after 8 days of exposure to the same toxin concentration [22]. Moreover, a 10% inhibition of *Medicago sativa* germination was verified after exposure to cyanobacterial bloom material containing 2200 μg/L MCs [24]. Decrease in plant growth, yield or photosynthetic efficiency was also reported in several agricultural crops exposed for prolonged periods (30 days) to cyanobacterial bloom material containing between 500 μg/L and 22,400 μg/L MCs [24,30]. On the other hand, little data has been reported so far regarding the effects of low toxin concentrations in plants. Chen *et al.* [33] reported that 10-day exposure to 10–200 μg/L MC-LR had no effect on root growth of rice seedlings. However, exposure to high toxin concentrations (2000–4000 μg/L) impeded rice root morphogenesis by inhibiting root elongation, crown root formation, and lateral root development. Moreover, studies also demonstrated that initial exposure to low concentrations (1–100 μg/L) of the toxin congener MC-RR could accelerate the germination of rape and cabbage [32]. However, extended exposure (e.g., 8 days) to 100 and 1000 μg/L MC-RR were toxic to seedlings. The authors also recognized that the exposure to MC-RR during the germination and seedling growth could compromise subsequent stages of development.

Still to be characterized is the impact of MCs in adult plants and the long term effects of exposure to low toxin concentrations, more commonly reported in water bodies and irrigation waters (e.g., 1–100 μg/L). In this regard, the present study highlights the changes in leaf metabolism consistent with the hypothesis of the impairment of the physiological condition of tomato plants. However, it was not possible to report morphological alterations and other physiological symptoms of stress, besides the decrease in chlorophyll fluorescence. One can thus raise the question of whether long term exposure leads to a boost in toxicity and provokes more adverse effects in plant growth and yield. To answer this, laboratory experiments that mimic chronic exposure may be considered in future studies; these may help to clarify the true impact of algal toxins in agriculture. We should also consider the fact that the availability of the toxin to the plants (not estimated in this study) might have been reduced to a certain extent due to interactions and adsorption to the clay aggregates. If less toxin is available for up-take, less toxic effects are expected.

Proteomics is an emergent research tool enabling the investigation of protein dynamics and variations in plant metabolism. Proteomics offers several advantages over the standard enzymatic and biochemical assays. It is sensitive to small variations in the expression of proteins and enables the characterization of posttranslational modifications [34]. Using 2DE and MALDI-TOF/TOF, we report several molecular markers that complement the current understanding of the mode of action of MC-LR in plants. MCs toxicity was previously related to oxidative stress and the activity of antioxidant defense enzymes, and the synthesis of the antioxidant metabolite tocopherol [21,27,28,33,35] or the inhibition of the activity of protein phosphatases [23,36]. Cyanotoxin treatment was shown to induce microtubule disruption in meristematic cells and to impair mitosis [23]. MCs were also shown to induce DNA damage in plants [37] and to induce changes in the activity of single-strand preferring nuclease (SSP nuclease) isoenzymes linking the early chromatin condensation and subsequent necrosis in *Phragmites australis plantlets* [38].

### 3.2. Bioaccumulation of MC-LR and Toxin Translocation from Roots to Leaves and Fruits

In the present work, MC-LR was detected in fruits, roots, and leaves of the tomato plant. The lowest concentrations were reported in green tomatoes (5.15–5.41 μg/kg fw). Increasingly higher concentrations were found in mature tomatoes (10.52–10.83 μg/kg fw), roots (1635.21 μg/kg fw), and leaves (27,673.21 μg/kg fw). Surprisingly, concentrations in fruits decreased to below LD (0.58 μg/L MC-LR) during the second week of the exposure experiment. This condition is not easy to explain in light of the current knowledge of cyanotoxin uptake. The necessity to obtain more information regarding the dynamics of toxin uptake and accumulation, and regarding the mechanisms of uptake, translocation, and toxin metabolization become evident. Some hypotheses can be brought to light, which can help understand this particular condition. For instance, the toxin can be chemically modified or metabolized in plant tissues after the second week. Supporting this hypothesis is the growing evidence that MCs are able to form conjugates with the peptide glutathione (GSH), spontaneously or catalyzed by the phase II xenobiotic metabolism, glutathione-S-transferase (GST) [39,40]. After 2 weeks of exposure, the toxin may also become inaccessible for quantification as a result of the binding to other biomolecules, e.g., protein phosphatases [41]. Furthermore, we may also consider that the toxin is deposited in the vacuole or in the apoplastic cell walls. This has been pointed out as a mechanism to protect cells from several endogenous and exogenous toxic compounds [42]. Moreover we cannot discard the hypothesis that the toxin simply is more diluted in the fruits due to an increase in the volume of the fruits and increase in water accumulation related to the fruit growth and maturation, from Week 1 to Week 2. In the present study, the detection of MCs in plant tissues was accomplished by solvent extraction, which is limited only to the free (*i.e.*, non-covalently bound) toxin [41]. 

This fraction may be the most important for monitoring and for food safety assessment since it is readily available and bioactive. The toxin was detected in the roots of tomato plants exposed to MCE, but not MCP. This condition may reflect the differential activity of the crude extract relative to the pure toxin, evidence has shown that crude cyanobacterial extracts induce molecular responses, which are different from the pure toxin and can have more severe consequences for the plants [28]. Moreover, the toxin concentration reported in tomato leaves was surprisingly high. This is likely the highest concentration reported in crops, which has been confirmed by ELISA test. We cannot definitively explain this result, however, we would suggest a putative role of evapotranspiration in this case. Evapotranspiration at the leaf level is the main motor of water movement from the soil across the plant. This process allows nutrient flow and is also considered to play a role in the uptake and accumulation of soil pollutants in plant tissues [42]. Both genetic and environmental factors (e.g., temperature, humidity, and light intensity) affect the transpiration rates. The leaves presenting higher transpiration rates, such as the young fully expanded and mature leaves [43,44], will likely tend to accumulate higher toxin concentrations. With the exception of the leaf tissues, all other *L. esculentum* tissues presented toxin concentrations within the range of concentrations reported previously. Exposure via roots of clover, rape, and lettuce plants, with lake water containing 2.1 mg/L total MCs during 15 days, led to the accumulation of 200–1450 μg/kg root dw [16]. However, Peuthert *et al.* [17] reported the accumulation of MCs in roots and shoots of seedlings of 11 agricultural crops after spray irrigation with 5 μg/L MC-LR or MC-LF for 24 h. In general, concentrations in the shoot occurred at a much lower level than in the root. The concentration of MC-LR in the roots of the seedlings varied between 12 and 125 μg/kg fw. Toxin concentrations measured in the shoots ranged from 4 to 44 μg/kg fw (MC-LR). The average bioconcentration factor (BCF) in shoots was 3.6 and in roots 8.4 [17]. Moreover, *Malus pumila* shoot cultures accumulated MCs up to 510.23 μg/kg fw, equivalent to an accumulation rate of 36.45 μg/kg day, after 14 days of exposure to 3000 μg/L total MCs [33]. On the other hand, different crops growing in the field and irrigated with contaminated groundwater (0.3–1.8 μg/L MCs) accumulated MCs. The highest toxin concentration was detected in radish (1200 μg/kg fw) and the lowest in cabbage (70 μg/kg fw). With the exception of dill, all other vegetables accumulated more toxins in the roots than in the leaves [15].

Food-basket surveys provide further evidence that MCs are accumulated in vegetable products destined for human consumption. In this regard, the toxin has been identified in water chestnuts (average values of 7 μg/kg dw) [13] and rice grains, 1.01–1.89 μg/kg and 2.05–3.19 μg/kg MC-LR in 15.9% and 9.1%, respectively, of total samples analyzed [37]. More recently Romero-Oliva *et al.* [14] reported 1.16 and 1.03 μg/kg dw total MCs in fruits of *L. esculentum* and *Capsicum annuum* field cultivated with contaminated lake water.

## 4. Experimental Section

### 4.1. Biological Material

Tomato plants (*Lycopersicon esculentum*) were grown hydroponically, in 4L pots containing expanded clay aggregate as a support medium for plants. Plants were watered every 3 days with adapted nutrient solution (500 mL/plant) containing Mg (50 mg/L), K (199 mg/L), P (62 mg/L), N (113 mg/L), Ca (122 mg/L), Fe (2.5 mg/L), B (0.44 mg/L), Cu (0.05 mg/L), Cl (0.85 mg/L), Mn (0.62 mg/L), Mo (0.06 mg/L), Zn (0.09 mg/L) [44]. Plants were grown in a greenhouse with natural light and photoperiod, from May to July 2011. *Microcystis aeruginosa* (LEGE 91094) strain was grown as described previously in bulk axenic cultures with medium Z8, at 25 °C, 22 μEm^−2^s^−1^ light intensity with a light/dark period of 14/10 h [45]. After 45 days of culture, the biomass was collected by centrifugation. Finally, the biomass was frozen at −80 °C and lyophilized (Telstar Cryodos 80 Model). *M. aeruginosa* strain LEGE 91094 produces MC-LR (95%) and low amounts of MC-LA and [D-Asp3]-MC-LR [46]. In this respect, in this work, we can consider that the toxic effects derived from this exposure are mainly to with the MC-LR bioactivity.

### 4.2. Cyanobacterial Cell Extracts and Quantification of MC-LR

*M. aeruginosa* extracts (MCE) were obtained according to the method described by Moreno *et al.* (2004) [47]. Filtrated cells (10.4 g) were mixed with distilled water, stirred during 20 min, and sonicated in a bath three times at room temperature. After that, cells were sonicated with Vibracell, 60 HZ, 5 × 1 min, with intervals of 1 min. The mixture was centrifuged at 20,000 × g during 20 min at 4 °C. The supernatant was collected and the extraction process was repeated once. The supernatants were thereafter pooled. The concentration of MC-LR in the extracts was estimated by high performance liquid chromatography (HPLC) with a photo diode array detector (HPLC-PDA) as described previously [48]. Toxin stock solutions were subsequently diluted to 1 μg/mL (MCE stock solution). MC-LR with 99% purity grade (Alexis, Lausen, Switzerland) was prepared in ultra pure water (Mili Q) to 1 μg/mL (MCP stock solution).

### 4.3. Experimental Exposure

The toxin exposure experiment was carried out using 2 month-old plants at the flowering and fruit maturation stage. Three experimental groups were performed, each group consisting of three plant replicates (*n* = 3). Group 1 (control, C) was exposed to the vehicle only (nutrient solution). Groups 2 and 3 were exposed to 0.5 L of nutrient solution containing, *M. aeruginosa* extracts with 100 μg/L MC-LR (MCE) or 100 μg/L of pure MC-LR (MCP), respectively. Toxin exposures were repeated every 3 days. After 1 and 2 weeks of exposure, leaves (second and third pairs counting from the apex), fruits (green and mature tomatoes), and roots (2nd week) from each plant were collected, the fresh weight determined and stored at −80 °C for subsequent analysis. Sampling of the roots after Week 1 was not performed since this would require the removal of the clay aggregate that supports the plants in the pots. This would involve additional stress to the plants and therefore was only performed at the end of the experiment.

### 4.4. MC-LR Extraction, Clean-up and Quantification in Plant Tissues

The fresh weight of plant tissues was determined and subjected to a first homogenization step with a blender (15 s cycles during 3 min.) on ice with 50% (v/v) methanol. A second homogenization step was performed with ultrasound and the free (unbound) MC-LR extracted, following the procedure described in Section 4.2 for the extraction of the toxin from cyanobacterial cells. MC-LR enriched fractions were obtained using Solid-Phase Extraction cartridges (SPE) (500 mg/6 mL C-18-E, Strata^®^ Phenomenex, CA, USA). After sample loading and column washing with 20% MeOH the toxin was eluted with 80% MeOH. MC-LR fractions were concentrated in vacuum in a rotary evaporator (Rotoquímica Büch, Switzerland) and the residue resuspended in 1 mL 50% MeOH prior to LC-MS/MS analysis. The LC-MS/MS system consisted of a LCQ Fleet ion trap MSn (ThermoScientific, Waltham, MA, USA) with electrospray (ESI) interface, including a Surveyor LC pump, a Surveyor auto sampler and a Surveyor photoelectric diode array (PDA) detector. Separation was achieved on C18 Hypersil Gold column (100 × 4.6 mm I.D., 5 μm, ThermoScientific, Waltham, MA, USA) or LiChrospher 100 RP-18 column (Merck, New Jersey, NJ, USA) kept at 25 °C, with a flow rate of 0.8 mL/min. The injected volume was 25 μL. A gradient elution was used with mobile phase A, methanol and B water, both were acidified with 0.1% formic acid. Mobile phase A was linearly increased from 55% to 90% in 12 min, then increased to 100% in 0.5 min and held for 2.5 min, finally brought back to 55% and held for 10 min until the next injection. Under these conditions the MC-LR retention time was 5.8 min. The mass spectrometer was operated in a multiple reaction monitoring mode (MRM) with a collision energy of 35 eV. The capillary voltage and tube lens were maintained at 22 and 120 V, respectively. Nitrogen was used as a sheath and auxiliary gas. Helium was used as a collision gas in the ion trap. The sheath gas flow rate was set at 80 (arbitrary units) and the capillary temperature was held at 350 °C. Samples were analyzed using the mass-to-charge ratio (*m*/*z*) transition of 995 > 599, at 23V collision energy. The MC-LR transition was monitored for 1 microscan time. MC-LR reference fragment ions with *m*/*z* values of 375, 553, 599, 866 and 977 were monitored in the MS/MS mode, as to validate the presence of the toxin in the plant tissues.

### 4.5. Chlorophyll Fluorescence

Chlorophyll fluorescence was determined through pulse amplitude modulation (PAM) fluorometry with PAM 2000 (Walz, Effeltrich, Germany). Second and third leaf pairs in the apex from dark-adapted plants were illuminated with a pulse of saturating light, and the fluorescence emitted was immediately recorded by the instrument. This procedure allows measuring the maximum fluorescence yield of Photosystem II (Fv/Fm), and it is directly related with the photosynthetic activity of the plant [49]. Two measurements were performed per plant replicate, one from control and on from the treated groups.

### 4.6. Protein Extraction for 2DE

Tomato leaves were ground in liquid nitrogen with a mortar and pestle. The ground tissue powder (0.1 g) was homogenized in cold acetone with TCA (10%, w/v) and mercaptoethanol (β-ME) (0.07%, v/v) for 1 h at −20 °C. The homogenate was centrifuged and the pellet washed with cold acetone with β-ME (0.07%) [50]. The protein pellet was subsequently dried at room temperature and solubilized for 1 h in 250 μL of urea (7 M), thiourea (2 M), 3-[(3-cholamidopropyl)dimethylammonio]-1-propane sulfonate (CHAPS) (4%, w/v), dithiothreitol (65 mM) and ampholytes, pH 4–7 (0.8%, v/v) (protein solubilization buffer, SB). The homogenate was centrifuged at 16,000 ×g, for 20 min at 22 °C. The supernatant was collected and proteins quantified with the Bradford method [51]. Protein samples were stored at −80 °C.

### 4.7. Two-Dimensional Gel Electrophoresis (2DE)

The two-dimensional gel electrophoresis was based in the procedure described by Campos *et al.* [50]. Protein samples (300 μg of protein) were diluted to 300 μL in SB buffer. Diluted samples were loaded in 17 cm, pH 4–7 IEF gel strips (Bio-Rad, Hercules, CA, USA) and proteins separated by isoelectric focusing (IEF) in a Protean IEF Cell (Bio-Rad, Hercules, CA, USA) with the following program—16 h at 50 V (strip rehydration); Step 1, 15 min at 250 V; Step 2, 3 h voltage gradient to 10,000 V (linear ramp); Step 3, 10,000 V until achieving 60,000 V/h (linear ramp). Paper wicks were used in the electrodes to remove the excess of salts from the samples. After the first dimension IEF gel strips were stored at −20 °C until performing the second-dimension sodium dodecyl sulfate polyacrylamide gel electrophoresis (SDS-PAGE). IEF gel strips were equilibrated using 10 mg/mL dithiothreitol and 25 mg/mL iodoacetamide in urea (6 M), glycerol (30%, v/v), and SDS (2%, w/v) [50]. Subsequently, IEF gel strips were assembled on top of 12% (w/v) acrylamide SDS-PAGE slab gels (20 cm × 20 cm × 1 cm) and proteins separated by SDS-PAGE in a Protean Xi Cell (Bio-Rad, Hercules, CA, USA) at 24 mA per gel. In this procedure, one 2DE gel was run per replicate. Colloidal coomassie blue was used for gel staining and protein visualization [52].

### 4.8. Gel Image Acquisition and Protein Expression Analysis

2DE gel images were acquired using a calibrated scanner (GS-800, Bio-Rad, Hercules, CA, USA) and protein spots detected automatically with the PDQuest 2-D Analysis Software (Bio-Rad, Hercules, CA, USA) [53]. Sensitivity parameters were reproduced for every gel image. Spot detection and spot matching was manually revised in the software and spot intensities normalized in terms of the total density in the gel image. For protein expression analysis, a master gel was obtained in the software with all the spots detected in the 2DE gel images. The presence/absence of spots and quantitative variations in spot intensities was subsequently analyzed by comparing the intensity of each protein spot between the experimental groups [53]. Quantitative variations were statistically validated using t-Student test (*p* ≤ 0.05).

### 4.9. Protein Identification

Protein spots were excised from gels and proteins subjected to in-gel digestion using the protease trypsin [54]. The tryptic digests were desalted and concentrated using reversed phase micro-columns [55]. The peptides were eluted directly onto the MALDI plate using the matrix α-cyano-4- hydroxycinamic acid (5 mg/mL) prepared in acetonitrile (70%, v/v) and trifluoroacetic acid (0.1%, v/v). Protein identification was done by MALDI-TOF-TOF with an Applied Biosystem 5800 Proteomics Analyzer (Applied Biosystems, Foster City, CA, USA) in MS and MS/MS mode. Spectra were externally calibrated using des-Arg-Bradykinin (904.468 Da), angiotensin 1 (1296.685 Da), Glu-Fibrinopeptide B (1570.677 Da), ACTH (1-17) (2093.087 Da), and ACTH (18-39) (2465.199 Da) (Calibration Mix from 4700 Applied Biosystems). Ten s/n best precursors from each MS spectrum were selected for MS/MS analysis. The generated mass spectra were used to search the National Center for Biotechnology Information (NCBI) predicted protein database restricted to “viridiplantae” and an eudicot EST database with the algorithm Molecular Weight Search (MOWSE), from MASCOT server 2.3 (Matrix-Science). Protein scores above 73 (*p* < 0.05) for proteins and 49 for individual peptide ion scores in MOWSE were considered as confident protein identification. The Mascot analysis of results was performed using the following parameters: missed-cleavage, two; peptide tolerance, 100 ppm; fragment mass: 0.5Da; fixed modifications: Carbamidomethyl (C); variable modifications: Dioxidation (W), Oxidation (HW), Oxidation (M), Trp- > Kynurenin (W).

### 4.10. Statistics

Data are expressed as mean ± standard deviation of three plants per group (*n* = 3). Statistical analysis was performed by analysis of variance (ANOVA) using GraphPad InStat software (GraphPad Software Inc., La Jolla, CA, USA). When differences were significant, Tukey’s test was used to compare the individual treatments. Statistical significance was inferred at *p* < 0.05.

## 5. Conclusions

In this work, adult tomato plants were exposed for 15 days to pure MC-LR or MC-LR in cyanobacterial extracts, followed by a proteomical investigation of the alterations in leaf metabolism and of the toxin accumulation in the different plant organs. Although no morphological and physiological alterations were observed in adult plants, the proteomic data anticipate a relevant switch-over of the metabolism with putative consequences to the physiology of the plants. Moreover, the toxin was detected in roots, fruits, and leaves, suggesting that it is taken up by the plant and is transported to the different organs. Taking as reference the tolerable daily intake established by the WHO (0.04 μg/kg body weight/day), the specific toxin concentrations reported in fruits could represent a health risk for consumers. In light of the present results, we recommend a careful monitoring of MC bioaccumulation in crop production systems affected by this source of contamination.

## Supplementary Files

Supplementary File 1 Supplementary Information (PDF, 137 KB)

## References

[1] Prieto A.I., Pichardo S., Jos A., Moreno I., Cameán A.M. (2007). Time dependent oxidative stress responses after acute exposure to toxic cyanobacterial cells containing microcystins in tilapia fish (*Oreochromis niloticus*) under laboratory conditions. Aquat. Toxicol..

[2] Gurbuz F., Metcalf J.S., Karahan A.G., Codd G.A. (2009). Analysis of dissolved microcystins in surface water samples from Kovada Lake, Turkey. Sci. Total Environ..

[3] Bartram J., Carmichael W.W., Chorus I., Jones G., Skulberg O.S., Chorus I., Bartram J. (1999). Introduction. Toxic cyanobacteria in Water: A Guide to their Public Health Consequences, Monitoring and Management.

[4] Sivonen K., Jones G., Chorus I., Bartram J. (1999). Cyanobacterial toxins. Toxic Cyanobacteria in Water: A Guide to their Public Health Consequences, Monitoring and Management.

[5] Wei Y., Weng D., Li F., Zou X., Young D.O., Ji J., Shen P. (2008). Involvement of JNK regulation in oxidative stress-mediated murine liver injury by microcystin-LR. Apoptosis.

[6] Puerto M., Gutiérrez-Praena D., Prieto A.I., Pichardo S., Jos A., Miguel-Carrasco J.L., Vázquez C.M., Cameán A.M. (2011). Subchronic effects of cyanobacterial cells on the transcription of antioxidant enzyme genes in tilapia (*Oreochromis niloticus*). Ecotoxicology.

[7] Pichardo S., Jos A., Zurita J.L., Salguero M., Cameán A.M., Repetto G. (2007). Acute and subacute toxic effects produced by microcystin-YR on the fish cell lines RTG-2 and PLHC-1. Toxicol. In Vitro.

[8] Puerto M., Pichardo S., Jos A., Cameán A.M. (2010). Microcystin-LR induces toxic effects in differentiated and undifferentiated Caco-2 cells. Arch. Toxicol..

[9] (2010). International Agency for Research on Cancer (IARC) monographs on the evaluation of carcinogenic risks to humans. Ingested nitrate and nitrite, and cyanobacterial peptide toxins. IARC Monogr. Eval. Carcinog. Risks Hum..

[10] Atencio L., Moreno I., Prieto A.I., Moyano R., Molina A.M., Cameán A.M. (2008). Acute effects of Microcystins MC-LR and MC-RR on acid and alkaline phosphatase activities and pathological changes in intraperitoneally exposed tilapia fish (*Oreochromis* sp.). Toxicol. Pathol..

[11] Codd G., Bell S., Kaya K., Ward C., Beattie K., Metcalf J. (1999). Cyanobacterial toxins, exposure routes and human health. Eur. J. Phycol..

[12] Zurawell R.W., Chen H.R., Burke J.M., Prepas E.E. (2005). patotoxic cyanobacteria: A review of the biological importance of microcystins in freshwater environments. J. Toxicol. Environ. Health B Crit. Rev..

[13] Xiao F.G., Zhao X.L., Tang J., Gu X.H., Zhang J.P., Niu W.M. (2009). Necessity of screening water chestnuts for microcystins after cyanobacterial blooms break out. Arch. Environ. Contam. Toxicol..

[14] Romero-Oliva C.S., Contardo-Jara V., Block T., Pflugmacher S. (2014). Accumulation of microcystin congeners in different aquatic plants and crops—A case study from lake Amatitlán, Guatemala. Ecotoxicol. Environ. Saf..

[15] Mohamed Z.A., Al Shehri A.M. (2009). Microcystins in groundwater wells and their accumulation in vegetable plants irrigated with contaminated waters in Saudi Arabia. J. Hazard. Mater..

[16] Crush J.R., Briggs L.R., Sprosen J.M., Nichols S.N. (2008). Effect of irrigation with lake water containing microcystins on microcystin content and growth of ryegrass, clover, rape, and lettuce. Environ. Toxicol..

[17] Peuthert A., Chakrabarti S., Pflugmacher S. (2007). Uptake of microcystins-LR and -LF (cyanobacterial toxins) in seedlings of several important agricultural plant species and the correlation with cellular damage (lipid peroxidation). Environ. Toxicol..

[18] Kós P., Gorzó G., Surányi G., Borbély G. (1995). Simple and efficient method for isolation and measurement of cyanobacterial hepatotoxins by plant tests (*Sinapis alba* L.). Anal. Biochem..

[19] Pereira S., Saker M.L., Vale M., Vasconcelos V.M. (2009). Comparison of sensitivity of grasses (*Lolium perenne* L. and *Festuca rubra* L.) and lettuce (*Lactuca sativa* L.) exposed to water contaminated with microcystins. Bull. Environ. Contam. Toxicol..

[20] El Khalloufi F., El Ghazali I., Saqrane S., Oufdou K., Vasconcelos V., Oudra B. (2012). Phytotoxic effects of a natural bloom extract containing microcystins on *Lycopersicon esculentum*. Ecotoxicol. Environ. Saf..

[21] Chen J., Song L., Dai J., Gan N., Liu Z. (2004). Effects of microcystins on the growth and the activity of superoxide dismutase and peroxidase of rape (*Brassica napus* L.) and rice (*Oryza sativa* L.). Toxicon.

[22] Saqrane S., El Ghazali I., Oudra B., Bouarab L., Vasconcelos V. (2008). Effects of cyanobacteria producing microcystins on seed germination and seedling growth of several agricultural plants. J. Environ. Sci. Health B.

[23] Máthé C., Beyer D., Erdodi F., Serfozo Z., Székvölgyi L., Vasas G., M-Hamvas M., Jámbrik K., Gonda S., Kiss A. (2009). Microcystin-LR induces abnormal root development by altering microtubule organization in tissue-cultured common reed (*Phragmites australis*) plantlets. Aquat. Toxicol..

[24] El Khalloufi F., Oufdou K., Lahrouni M., El Ghazali I., Saqrane S., Vasconcelos V., Oudra B. (2011). Allelopatic effects of cyanobacteria extracts containing microcystins on *Medicago sativa-Rhizobia* symbiosis. Ecotoxicol. Environ. Saf..

[25] Lahrouni M., Oufdou K., Faghire M., Peix A., El Khalloufi F., Vasconcelos V., Oudra B. (2012). Cyanobacterial extracts containing microcystins affect the growth, nodulation process and nitrogen uptake of faba bean (*Vicia faba* L., Fabaceae). Ecotoxicology.

[26] Smith R.D., Walker J.C. (1996). Plant protein phosphatases. Annu. Rev. Plant Physiol. Plant Mol. Biol..

[27] Pflugmacher S., Hofmann J., Hübner B. (2007). Effects on growth and physiological parameters in wheat (*Triticum aestivum* L.) grown in soil and irrigated with cyanobacterial toxin contaminated water. Environ. Toxicol. Chem..

[28] Peuthert A., Pflugmacher S. (2010). Influence of the cyanotoxin microcystin-LR on tocopherol in Alfalfa seedlings (*Medicago sativa*). Toxicon.

[29] Prieto A., Campos A., Cameán A., Vasconcelos V. (2011). Effects on growth and oxidative stress status of rice plants (*Oryza sativa*) exposed to two extracts of toxin-producing cyanobacteria (*Aphanizomenon ovalisporum* and *Microcystis aeruginosa*). Ecotoxicol. Environ. Saf..

[30] Saqrane S., Ouahid Y., El Ghazali I., Oudra B., Bouarab L., del Campo F.F. (2009). Physiological changes in *Triticum durum*, *Zea mays*, *Pisum sativum* and *Lens esculenta* cultivars, caused by irrigation with water contaminated with microcystins: A laboratory experimental approach. Toxicon.

[31] Saqrane S., Ghazali I.E., Ouahid Y., Hassni M.E., Hadrami I.E., Bouarab L., del Campo F.F., Oudra B., Vasconcelos V. (2007). Phytotoxic effects of cyanobacteria extract on the aquatic plant *Lemna gibba*: Microcystin accumulation, detoxication and oxidative stress induction. Aquat. Toxicol..

[32] Bibo L., Yan G., Bangding X., Jiantong L., Yongding L. (2008). A laboratory study on risk assessment of microcystin-RR in cropland. J. Environ. Manage..

[33] Chen J., Dai J., Zhang H., Wang C., Zhou G., Han Z., Liu Z. (2010). Bioaccumulation of microcystin and its oxidative stress in the apple (*Malus pumila*). Ecotoxicology.

[34] Campos A., Tedesco S., Vasconcelos V., Cristobal S. (2012). Proteomic research in bivalves: Towards the identification of molecular markers of aquatic pollution. J. Proteomics.

[35] Yin L., Huang J., Huang W., Li D., Wang G., Liu Y. (2005). Microcystin-RR-induced accumulation of reactive oxygen species and alteration of antioxidant systems in tobacco BY-2 cells. Toxicon.

[36] Peuthert A., Lawton L., Pflugmacher S. (2008). *In vivo* influence of cyanobacterial toxins on enzyme activity and gene expression of protein phosphatases in Alfalfa (*Medicago sativa*). Toxicon.

[37] Chen J., Han F.X., Wang F., Zhang H., Shi Z. (2012). Accumulation and phytotoxicity of microcystin-LR in rice (*Oryza sativa*). Ecotoxicol. Environ. Saf..

[38] Jámbrik K., Máthé C., Vasas G., Beyer D., Molnár E., Borbély G., M-Hamvas M. (2011). Microcystin-LR induces chromatin alterations and modulates neutral single-strand-preferring nuclease activity in *Phragmites australis*. J. Plant Physiol..

[39] Buratti F.M., Scardala S., Funari E., Testai E. (2013). The conjugation of microcystin-RR by human recombinant GSTs and hepatic cytosol. Toxicol. Lett..

[40] Setlíková I., Wiegand C. (2009). Hepatic and branchial glutathione S-transferases of two fish species: Substrate specificity and biotransformation of microcystin-LR. Comp. Biochem. Physiol. C Toxicol. Pharmacol..

[41] Suchy P., Berry J. (2012). Detection of total microcystin in fish tissues based on lemieux oxidation, and recovery of 2-methyl-3-methoxy-4-phenylbutanoic acid (MMPB) by solid-phase microextraction gas chromatography-mass spectrometry (SPME-GC/MS). Int. J. Environ. Anal. Chem..

[42] Trapp S., Eggen T. (2013). Simulation of the plant uptake of organophosphates and other emerging pollutants for greenhouse experiments and field conditions. Environ. Sci. Pollut. Res. Int..

[43] Václavík J. (1973). Effect of different leaf age on the relationship between the CO_2_ uptake and water vapour efflux in tobacco plants. Biol. Plant..

[44] Constable G.A., Rawson H.M. (1980). Effect of leaf position, expansion and age on photosynthesis, transpiration and water use efficiency of cotton. Aust. J. Plant Physiol..

[45] Saker M.L., Nogueira I.C., Vasconcelos V.M., Neilan B.A., Eaglesham G.K., Pereira P. (2003). First report and toxicological assessment of the cyanobacterium *Cylindrospermopsis raciborskii* from Portuguese freshwaters. Ecotoxicol. Environ. Saf..

[46] Vasconcelos V.M. (1995). Uptake and depuration of the heptapeptide toxin microcystin-LR in *Mytilus galloprovincialis*. Aquat. Toxicol..

[47] Moreno I.M., Pereira P., Franca S., Cameán A. (2004). Toxic cyanobacteria strains isolated from blooms in the Guadiana river (southwestern Spain). Biol. Res..

[48] Pinheiro C., Azevedo J., Campos A., Loureiro S., Vasconcelos V. (2013). Absence of negative allelopathic effects of cylindrospermopsin and microcystin-LR in selected marine and freshwater phytoplankton species. Hydrobiologia.

[49] Maxwell K., Johnson G.N. (2000). Chlorophyll fluorescence—A practical guide. J. Exp. Bot..

[50] Campos A., Da Costa G., Coelho A.V., Fevereiro P. (2009). Identification of bacterial protein markers and enolase as a plant response protein in the infection of *Olea europaea* subsp. europaea by *Pseudomonas savastanoi* pv. Savastanoi. Eur. J. Plant Pathol..

[51] Bradford M.M. (1976). A rapid and sensitive method for the quantitation of microgram quantities of protein utilizing the principle of protein-dye binding. Anal. Biochem..

[52] Neuhoff V., Arold N., Taube D., Ehrhardt W. (1988). Improved staining of proteins in polyacrylamide gels including isoelectric focusing gels with clear background at nanogram sensitivity using Coomassie Brilliant Blue G-250 and R-250. Electrophoresis.

[53] Puerto M., Campos A., Prieto A., Cameán A., de Almeida A.M., Coelho A.V., Vasconcelos V. (2011). Differential protein expression in two bivalve species; *Mytilus galloprovincialis* and *Corbicula fluminea*; exposed to Cylindrospermopsis raciborskii cells. Aquat. Toxicol..

[54] Pandey A., Mann M. (2000). Proteomics to study genes and genomes. Nature.

[55] Gobom J., Nordhoff E., Mirgorodskaya E., Ekman R., Roepstorff P. (1999). Sample purification and preparation technique based on nano-scale reversed-phase columns for the sensitive analysis of complex peptide mixtures by matrix-assisted laser desorption/ionization mass spectrometry. J. Mass Spectrom..

