# Supplementary material for: Exposure of Lycopersicon Esculentum to Microcystin-LR: Effects in the Leaf Proteome and Toxin Translocation from Water to Leaves and Fruits
Source: Toxins (Basel). 2014 Jun 11;6(6):1837–54. doi: 10.3390/toxins6061837 (PMC4073132; doi:10.3390/toxins6061837)
Supplement: Supplementary File 1 — Supplementary Information (PDF, 137 KB) [file toxins-06-01837-s001.pdf]

Supplementary Information

Figure S1. LC-MS and MS/MS analysis of MC-LR in the different tissues of adult Lycopersicon esculentum plants, roots (**a**); green tomatoes (**b**); red tomatoes (**c**) and leaves (**d**). MC-LR reference fragment ions (#).

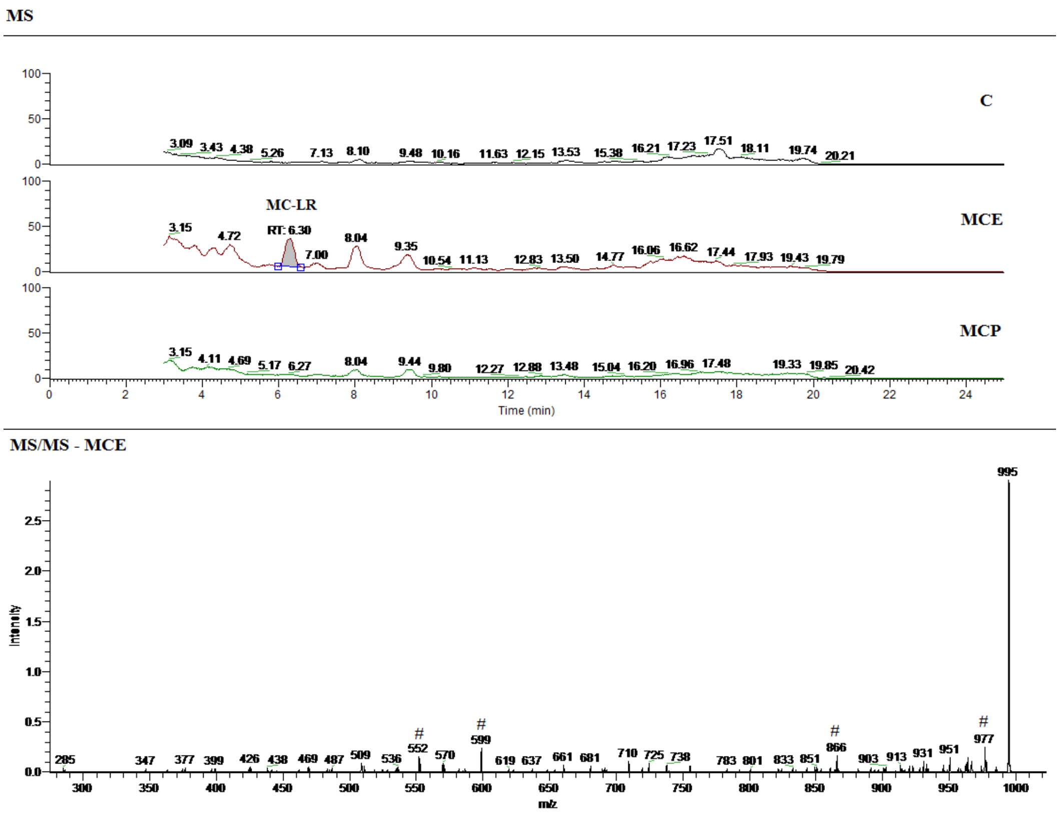

(**a**)

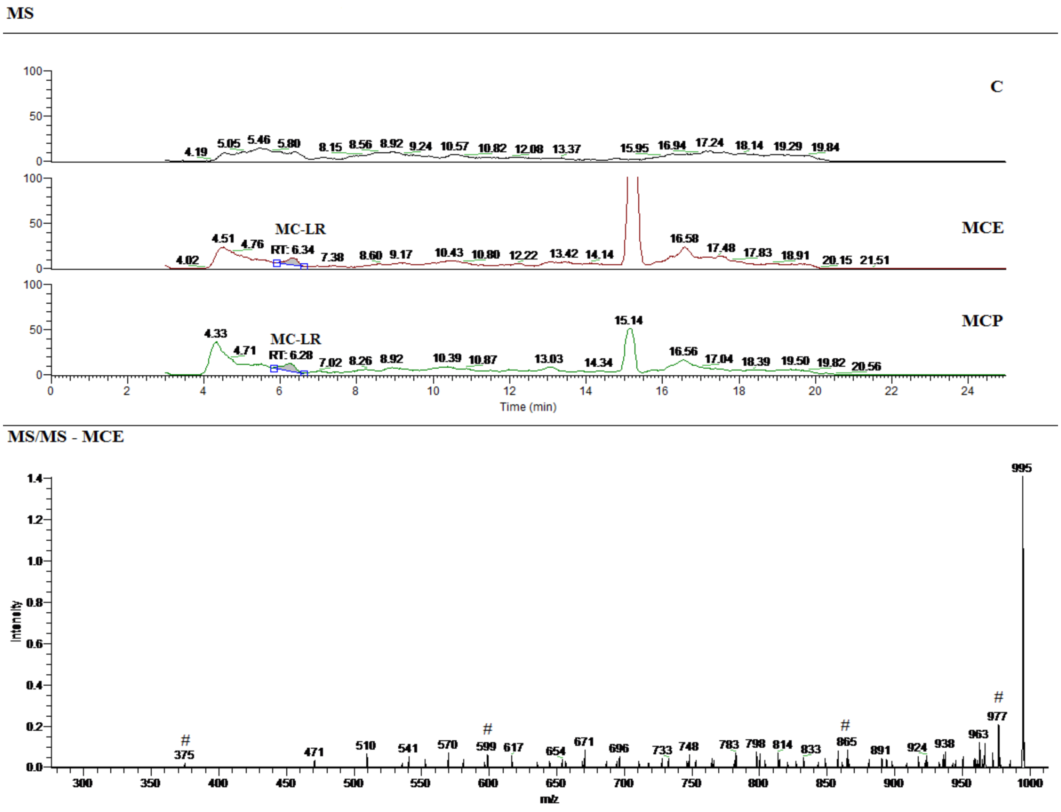

(**b**)

Figure S1. *Cont.*

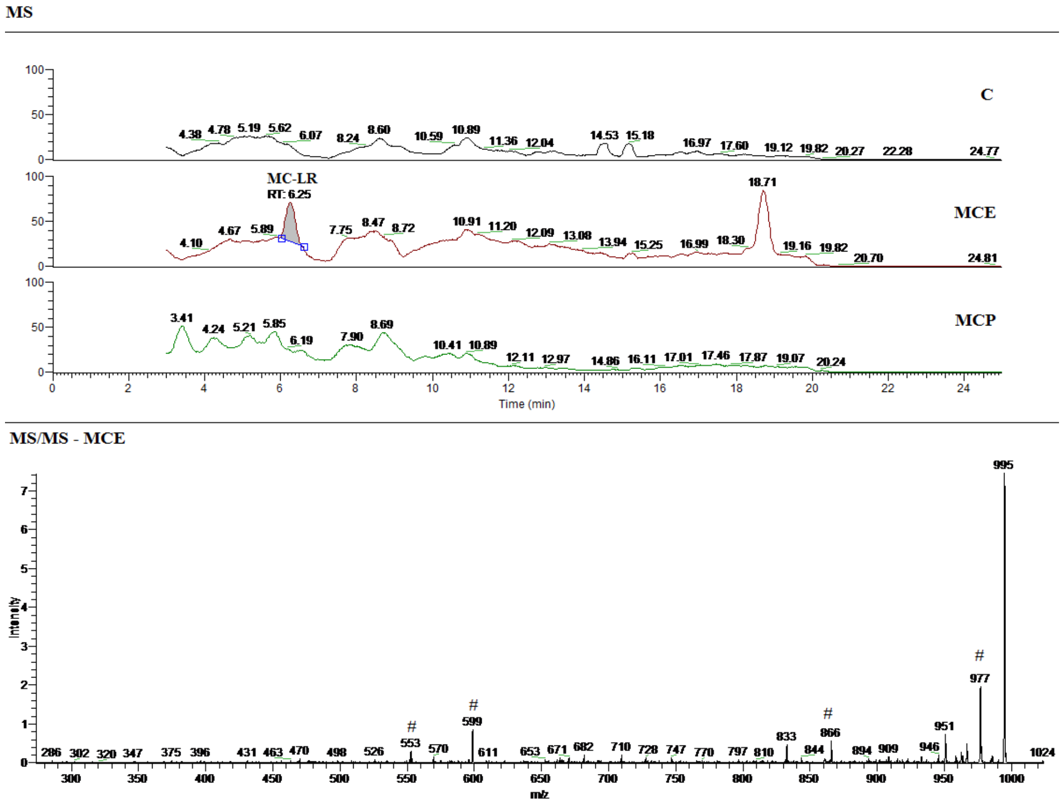

(c)

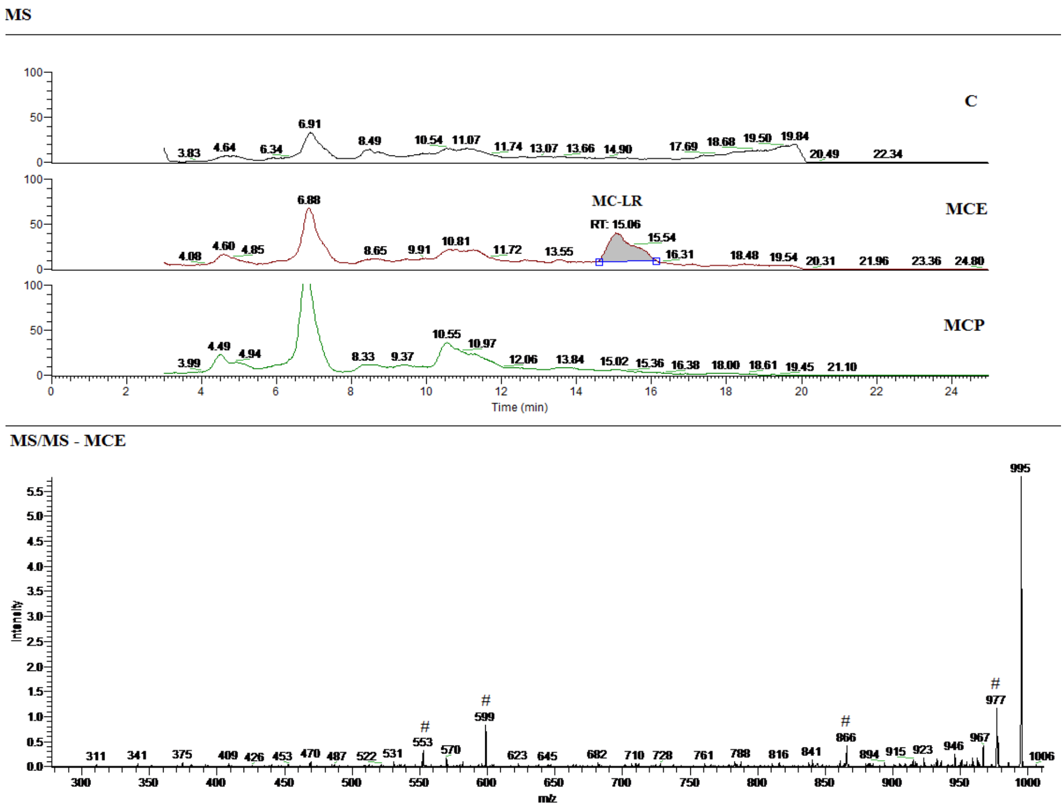

(d)
